# Parental postpartum depression directly and indirectly affects exclusive breastfeeding: a cross-sectional study

**DOI:** 10.3389/fpubh.2026.1724216

**Published:** 2026-02-04

**Authors:** Yuefeng Tan, Xiaona Na, Lei Yu, Sitong Luo, Ai Zhao

**Affiliations:** 1 Vanke School of Public Health, Tsinghua University, Beijing, China; 2 Lanzhou Maternal and Child Health Care Hospital, Lanzhou, Gansu, China

**Keywords:** postpartum depression, exclusive breastfeeding, maternal intention, path analysis, parental mental health

## Abstract

**Background:**

Exclusive breastfeeding (EBF) is essential for infant health. Parental postpartum depression (PPD) may play a crucial role in shaping maternal intention and behavior to breastfeeding. This study, grounded in the ABC model of behavior theory, aimed to examine the direct and indirect pathways linking maternal and paternal PPD with EBF intention and practice.

**Methods:**

A total of 273 couples attending postpartum health check-ups were invited to complete structured questionnaires with assistance from trained interviewers. Symptoms of PPD in both mothers and fathers were assessed using the Edinburgh Postnatal Depression Scale (EPDS). Logistic regression analyses were conducted to examine the associations between parental PPD and intention and practice related to EBF. Path analysis was further employed to identify potential pathways linking parental PPD, maternal intention to EBF, and EBF practice. Direct and indirect effects were estimated within the path model using the weighted least squares mean and variance adjusted (WLSMV) estimator. Standardized path coefficients (*β*) were reported for all effects.

**Results:**

Among 273 couples, 41.0% of mothers and 28.5% of fathers screened positive for PPD. Although most mothers (93.4%) showed a positive intention to EBF, only 52.4% practiced it. Maternal depression was associated with a less positive intention to EBF (adjusted OR = 0.20, 95% CI: 0.06–0.64). Path analysis further revealed that paternal PPD indirectly influenced maternal intention through maternal PPD (*β*_indirect_ = −0.177, *p* = 0.001), while maternal PPD exerted an indirect effect on EBF practice through intention (*β*_indirect_ = −0.189, *p* = 0.038). These findings highlight a family-level psychological pathway linking parental mental health to breastfeeding behaviors.

**Conclusion:**

A substantial gap was found between willingness and actual practice of EBF. Both maternal and paternal postpartum depression were directly and indirectly associated with breastfeeding intention and practice. Early identification and intervention for parental depression are essential to promote exclusive breastfeeding.

## Introduction

Extensive evidence has demonstrated the substantial benefits of breastfeeding for both mothers and infants. Breastfeeding contributes to lower maternal risks of cancers and mental disorders and reduces the likelihood of infectious diseases, obesity, diabetes, and other chronic conditions in children (1–3). Considered as the optimal feeding method, the WHO and UNICEF recommend that babies should be exclusively breastfed (EBF) for the first 6 months after birth, without adding any other food or liquids (4). Although the promotion of breastfeeding has been recognized as a global priority for improving child survival and health, only about 47% of infants under 6 months are exclusively breastfed worldwide, which remains far below the WHO’s target of at least 60% EBF within the first 6 months of life by 2030 (5).

In the exploration of factors associated with breastfeeding, postpartum depression (PPD) appears to be a potentially deleterious contributor (6–8). Approximately 17% of women experience PPD following childbirth (9). PPD usually occurs in women within 4–6 weeks of delivery, and has a long-lasting influence on maternal mental health. It may arise from a combination of hormonal changes, psychological adjustment to motherhood, less social support and unpleasant living conditions (10). Several studies have demonstrated that PPD is correlated with impairment of the mother–infant bond and less breastfeeding self-efficacy, which are considered as plausible reasons for the interruption of breastfeeding (11–13).

In fact, not only maternal but also paternal PPD is a concerning mental disorder. It is reported that the prevalence of paternal depression is approximately 8% (14). Paternal PPD has a negative effect on communication among family members, especially parent–infant interactions, which may cause adverse emotion and behaviors in children (15). However, evidence on the association between paternal depression and breastfeeding is still scarce. In addition, according to the ABC Theory of Emotion proposed by Albert Ellis, a negative behavior is not directly caused by the activating event but the wrong beliefs generated by the individual’s incorrect cognition and evaluation about it (16). The intention to EBF, which was less mentioned in previous studies, may play a critical role in promoting EBF.

Therefore, this study aimed to investigate (i) association between maternal PPD and intention and practice of EBF; (ii) association between paternal PPD and intention and practice of EBF; and (iii) to perform a path analysis exploring the potential pathways linking paternal and maternal PPD, maternal intention to EBF, and breastfeeding practice.

## Materials and methods

### Participants

This cross-sectional study was conducted in Shenyang and Xuchang in China. In each city, one maternity and childcare hospital was selected by convenience. Infants’ parents who visited hospital for a postpartum health check-up during March to April 2021 were invited to participate in this study by local medical professionals. The eligible participants were women and their partners aged 18–45 years who had given birth to a single full-term infant within the past 3 months. The exclusion criteria included: (i) women not residing in the selected cities; (ii) women or infants with diseases requiring hospitalization or special medical care; (iii) parents with a history of psychological illness; and (iv) those with alcohol or drug addiction. A total of 530 mothers were invited to participate. After excluding cases with missing data, a total of 273 couples were included in the final analysis.

### Ethics and consent statement

The research protocol was approved by the Ethics Committee of Tsinghua University (THUSM/PHREC/2020400-005). All procedures were conducted in accordance with the Declaration of Helsinki. Written informed consent was obtained from all participants prior to the commencement of the study.

### Data collection

With the assistance of trained surveyors, participants completed a structured questionnaire comprising four sections: (i) socio-demographic characteristics; (ii) maternity-related information and postpartum lifestyle; (iii) breastfeeding intention and practice; and (iv) mental health assessment. For socio-demographic characteristics, information such as age, residence, education and the household size were collected. Maternity information included pregnancy and delivery history, and the current delivery information. The definition of exclusive breastfeeding used in this study was consistent with the WHO criteria, as stated in the Introduction, and this definition was clearly stated in the questionnaire and explained to participants by trained field researchers at the study site. Regarding breastfeeding intention to EBF were investigated by the question “Would you like to exclusive breastfeeding?” and participants could respond Yes or No. Actual feeding practice were investigated by the question “How do you feed your baby now?” and participants could choose the appropriate option from exclusive breastfeeding (breastfeeding only), mixed feeding, and other feeding methods. Participants who chose “breastfeeding only” were classified as currently practicing exclusive breastfeeding. Questions such as “Have you ever received professional guidance of breastfeeding?” were also asked. For the measurement of depression, the Edinburgh Postnatal Depression Scale (EPDS) was applied for parents of infants. The EPDS is a 10-item 4-point Likert scale used as a PPD screen, with a higher total score indicating higher risk of PPD. In this study, the Cronbach alpha was 0.81 (wife) and 0.82 (husband) respectively. A cut-off score of 10 is recommended as a Chinese PPD diagnosis by researchers for both male and female population (17–19). Spousal support was measured using a single self-reported item that asked mothers: “How much support and care did you receive from your husband during the breastfeeding period?” Participants selected one of four response options: none, very little, moderate, or full support. We categorized spousal support into three levels: low (none or very little), medium (moderate), and high (full support). All items including those of a sensitive nature, were completed independently by participants in a private setting, with husbands and wives answering the survey separately to ensure comfort and confidentiality.

### Statistical analysis

The RStudio (version 2023.09.1, Build 494; RStudio, PBC, Boston, MA, United States). and Mplus Version 7.4 (Muthén & Muthén Inc., Los Angeles, CA, United States) software were used for data analysis. The chi-square test and Fisher’s exact test were used to compare the distribution of EBF intention and practice among women with different characteristics, with the statistical significance level set at 0.05. Variables with significant results in the univariable analysis were considered as potential confounders and adjusted in the logistic regression analysis to examine the association between depression and EBF intention and practice. A path analysis model was established to explore the potential pathways linking parental PPD, maternal intention to EBF, and breastfeeding practice, using the weighted least squares mean and variance adjusted (WLSMV) estimator. Models with different pathways were established to test possible direct and indirect effects. Standardized path coefficients (*β*) were reported for all effects. The best-fitting model was selected based on comparative fit indices.

## Results

### Basic information of participants

The postpartum duration of participating mothers ranged from 0 to 72 days, with a mean of 15.9 ± 16.8 days. All newborns had an Apgar score of 10. None of the mothers smoked or consumed alcohol during pregnancy. Table 1 presents the socio-demographic characteristics and maternity history of the participating mothers, categorized by their intention and practice to EBF. Approximately 93.4% of mothers expressed a preference for EBF, while only 52.4% practiced it. Compared with mothers who had a negative intention to EBF, mothers with a positive intention to EBF had a higher proportion of urban residence, higher education levels, receipt of professional breastfeeding guidance, and stronger spousal support, as well as a lower prevalence of PPD and a higher prevalence of EBF practice. Among mothers who practiced EBF, a higher proportion lived in smaller households and had received feeding guidance from healthcare professionals compared with those who did not practice EBF. Table 2 displays the socio-demographic characteristics and maternity history of the participating mothers, categorized by levels of maternal and paternal PPD status. Using the EPDS, 41.0% of mothers and 28.5% of fathers were identified as having probable PPD. Mothers with PPD had a higher proportion living in smaller households and a higher prevalence of paternal PPD compared with mothers without PPD.

**Table 1:** Distribution of EBF intention and practice by maternal socio-demographic and maternity-related factors.

Factors	Intention to EBF				*P*	Practice of EBF				*P*
	Negative		Positive			No		Yes		
	*n*	%	*n*	%		*n*	%	*n*	%	
Overall	18	6.6	255	93.4	–	143	47.6	130	52.4	–
Age (y)										
Under 30	12	66.7	141	55.3	0.488	63	48.5	90	62.9	0.022^*^
30 and above	6	33.3	114	44.7		67	51.5	53	37.1	
Residence										
Rural	12	66.7	86	33.7	0.010^*^	51	39.2	47	32.9	0.351
Urban	6	33.3	169	66.3		79	60.8	96	67.1	
Education level										
No bachelor’s degree	13	72.2	105	41.2	0.020^*^	63	48.5	55	38.5	0.123
Bachelor’s degree	5	27.8	150	58.8		67	51.5	88	61.5	
Mode of delivery										
Vaginal delivery	13	72.2	182	71.4	1.000	87	66.9	108	75.5	0.151
Cesarean section	5	27.8	73	28.6		43	33.1	35	24.5	
Gravidity										
Multigravida	10	55.6	135	52.9	1.000	72	55.4	73	51.0	0.551
Primigravida	8	44.4	120	47.1		58	44.6	70	49.0	
Parity										
Multiparous	7	38.9	104	40.8	1.000	59	45.4	52	36.4	0.164
Primiparous	11	61.1	151	59.2		71	54.6	91	63.6	
Household size										
Small household (2)	3	16.7	69	27.1	0.417	24	18.5	48	33.6	0.007^*^
Large household (>2)	15	83.3	186	72.9		106	81.5	95	66.4	
Guidance of breastfeeding										
No	14	77.8	114	44.7	0.013^*^	78	60.0	50	35.0	0.001^*^
Yes	4	22.2	141	55.3		52	40.0	93	65.0	
Spousal support										
Low	2	11.1	14	5.5	0.020^*^	9	6.9	7	4.9	0.589
Medium	6	33.3	32	12.5		20	15.4	18	12.6	
High	10	55.6	209	82.0		101	77.7	118	82.5	
Maternal PPD										
Yes	12	66.7	100	39.2	0.041^*^	52	40.0	60	42.0	0.837
No	6	33.3	155	60.8		78	60.0	83	58.0	
Paternal PPD										
Yes	4	22.2	74	29.0	0.729	36	27.7	42	29.4	0.863
No	14	77.8	181	71.0		94	72.3	101	70.6	
Intention to EBF										
Negative	–	–	–	–	–	16	12.3	2	1.4	0.001^*^
Positive	–	–	–	–		114	87.7	141	98.6	
Practice of EBF										
No	16	88.9	114	44.7	<0.001^*^	–	–	–	–	–
Yes	2	11.1	141	55.3		–	–	–	–	

“–,” not applicable; *p* < 0.05 (^*^); All percentages represent column percentages.

**Table 2:** Distribution of maternal and paternal PPD status by maternal socio-demographic and maternity-related factors.

Factors	Maternal PPD				*P*	Paternal PPD				*P*
	No		Yes			No		Yes		
	*n*	%	*n*	%		*n*	%	*n*	%	
Overall	161	59.0	112	41.0	–	195	71.5	78	28.5	–
Age (y)										
Under 30	95	59.0	58	51.8	0.290	106	54.4	47	60.3	0.452
30 and above	66	41.0	54	48.2		89	45.6	31	39.7	
Residence										
Rural	60	37.3	38	33.9	0.662	64	32.8	34	43.6	0.125
Urban	101	62.7	74	66.1		131	67.2	44	56.4	
Education level										
No bachelor’s degree	73	45.3	45	40.2	0.470	89	45.6	29	37.2	0.254
Bachelor’s degree	88	54.7	67	59.8		106	54.4	49	62.8	
Mode of delivery										
Vaginal delivery	113	70.2	82	73.2	0.683	134	68.7	61	78.2	0.156
Cesarean section	48	29.8	30	26.8		61	31.3	17	21.8	
Gravidity										
Multigravida	81	50.3	64	57.1	0.322	102	52.3	43	55.1	0.774
Primigravida	80	49.7	48	42.9		93	47.7	35	44.9	
Parity										
Multiparous	61	37.9	50	44.6	0.321	84	43.1	27	34.6	0.250
Primiparous	100	62.1	62	55.4		111	56.9	51	65.4	
Household size										
Small household (2)	32	19.9	40	35.7	0.005^*^	56	28.7	16	20.5	0.216
Large household (>2)	129	80.1	72	64.3		139	71.3	62	79.5	
Guidance of breastfeeding										
No	78	48.4	50	44.6	0.620	94	48.2	34	43.6	0.578
Yes	83	51.6	62	55.4		101	51.8	44	56.4	
Spousal support										
Low	10	6.2	6	5.4	0.074	11	5.6	5	6.4	0.967
Medium	16	9.9	22	19.6		27	13.8	11	14.1	
High	135	83.9	84	75.0		157	80.5	62	79.5	
Intention to EBF										
Negative	6	3.7	12	10.7	0.041^*^	14	7.2	4	5.1	0.729
Positive	155	96.3	100	89.3		181	92.8	74	94.9	
Practice of EBF										
No	78	48.4	52	46.4	0.837	94	48.2	36	46.2	0.863
Yes	83	51.6	60	53.6		101	51.8	42	53.8	
Maternal PPD										
Yes	–	–	–	–	–	61	31.3	51	65.4	<0.001^*^
No	–	–	–	–		134	68.7	27	34.6	
Paternal PPD										
Yes	27	16.8	51	45.5	<0.001^*^	–	–	–	–	–
No	134	83.2	61	54.5		–	–	–	–	

“–,” not applicable; *p* < 0.05 (^*^); All percentages represent column percentages.

### Associations between parental PPD and maternal intention and practice to EBF

Table 3 presents the associations of maternal and paternal PPD with intention and practice of EBF. Mothers experiencing PPD were significantly less likely to hold a positive intention to EBF compared with mothers without PPD (adjusted OR = 0.20, 95% CI: 0.06–0.64). In contrast, paternal PPD was not significantly associated with maternal intention to EBF. Furthermore, neither maternal nor paternal PPD demonstrated a significant association with actual EBF behavior.

**Table 3:** Logistic regression analysis of the association between maternal and paternal PPD and intention and practice of EBF.

Variables	Intention to EBF (Positive)		Practice of EBF (Yes)	
	Crude OR (95%CI)	Adjusted OR (95%CI)	Crude OR (95%CI)	Adjusted OR (95%CI)
Maternal PPD status				
Yes	0.323^*^ (0.117 ~ 0.887)	0.201^*^ (0.063 ~ 0.644)	1.084 (0.669 ~ 1.758)	0.957 (0.560 ~ 1.635)
No	Ref	Ref	Ref	Ref
Paternal PPD status				
Yes	1.431 (0.456 ~ 4.490)	1.378 (0.408 ~ 4.648)	1.086 (0.641 ~ 1.838)	1.044 (0.590 ~ 1.847)
No	Ref	Ref	Ref	Ref

*p* < 0.05 (^*^). Adjusted ORs were adjusted for maternal age, residence, education level, household size, EBF guidance, and spousal support.

### Pathway analysis

Table 4 summarizes the correlations of the variables that were previously found to be significant predictors of EBF intentions and practice. In line with prior results, maternal depression was still linked to intention to EBF, but showed no significant relationship with actual practice. Paternal depression, on the other hand, showed no significant association with either EBF intention or practice. However, a significant positive correlation was observed between maternal intention to EBF and EBF practice. In addition, maternal PPD was significantly positively correlated with paternal PPD. Therefore, we further conducted a path analysis, as a form of structural equation modeling (SEM), to investigate whether parental depression indirectly influences EBF practice through its effect on maternal intention.

**Table 4:** Key variables and correlation coefficients (*Φ* or Cramer’s V).

Variables	Intention_*positive*	Practice_*EBF*	Age_*30*	Residence_*urban*	Education_*degree*	Household size_*larger*	Guidance_*yes*	Spousal support_*high*	Maternal PPD_*yes*	Paternal PPD_*yes*
Intention_*positive*	1.000	0.220*	0.060	0.170*	0.160*	−0.060	0.160*	0.167*	−0.140*	0.040
Practice_*EBF*	0.220*	1.000	−0.150*	0.070	0.100	−0.170*	0.250*	0.062	0.020	0.020
Age_*30*	0.060	−0.150*	1.000	0.220*	0.010	−0.090	0.090	0.125	0.070	−0.050
Residence_*urban*	0.170*	0.070	0.220*	1.000	0.270*	−0.150*	0.120	0.153*	0.030	−0.100
Education level_*degree*	0.160*	0.100	0.010	0.270*	1.000	−0.170*	0.100	0.185*	0.050	0.080
Household size_*larger*	−0.060	−0.170*	−0.090	−0.150*	−0.170*	1.000	−0.080	0.097	−0.180*	0.080
Guidance_*yes*	0.160*	0.250*	0.090	0.120	0.100	−0.080	1.000	0.135	0.040	0.040
Spousal support_*high*	0.167*	0.062	0.125	0.153*	0.185*	0.097	0.135	1.000	0.138	0.016
Maternal PPD_*yes*	−0.140*	0.020	0.070	0.030	0.050	−0.180*	0.040	0.138	1.000	0.310*
Paternal PPD_*yes*	0.040	0.020	−0.050	−0.100	0.080	0.080	0.040	0.016	0.310*	1.000

*p* < 0.05 (^*^).

**Figure 1:**
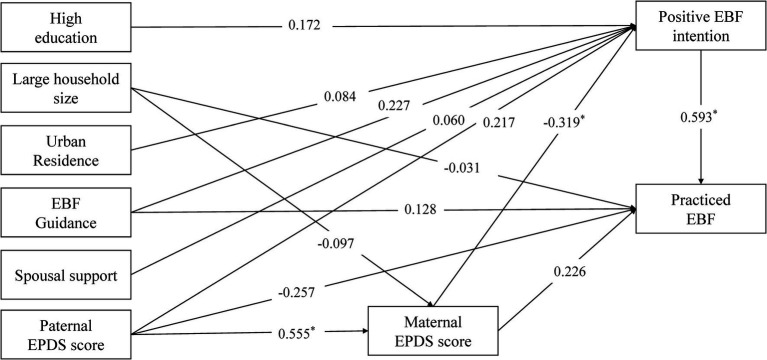
The pathway analysis model. *p* < 0.05 (^*^). Model fit index: RMSEA = 0.013, CFI = 0.997, TFI = 0.993, WRMR = 0.567.

The proposed model, as shown in Figure 1, demonstrated an excellent fit to the data: root mean square error of approximation (RMSEA) = 0.013, comparative fit index (CFI) = 0.997, Tucker–Lewis index (TLI) = 0.993, and weighted root mean square residual (WRMR) = 0.567. Maternal PPD was significantly negatively associated with maternal intention to EBF (*β*_total_ = −0.319, *p* = 0.001). Significant total effects were also observed between paternal PPD and maternal PPD (*β*_total_ = 0.555, *p* < 0.001) and between maternal EBF intention and EBF practice (*β*_total_ = 0.593, *p* = 0.001). In terms of indirect effects, paternal PPD significantly influenced maternal intention to EBF via maternal PPD (*β*_indirect_ = −0.177, *p* = 0.001), and maternal PPD had a significant indirect effect on EBF practice through maternal intention (*β*_indirect_ = −0.189, *p* = 0.038).

## Discussion

Our findings indicated a relatively higher prevalence of both willingness and practice of EBF. Notably, 93.4% of mothers expressed a positive intention to EBF, whereas only 52.4% practiced it, revealing a substantial gap between intention and behavior. This discrepancy may be explained by both family and professional factors. A higher proportion of mothers living in smaller households or those receiving professional breastfeeding guidance maintained EBF. Although larger households might provide emotional support and potentially reduce maternal depressive symptoms, excessive involvement from extended family members could interfere with consistent EBF practice. Meanwhile, guidance from healthcare professionals—such as home visiting and structured breastfeeding counseling—may help reinforce maternal confidence and promote adherence to EBF, which might partly explain why maternal PPD was not directly associated with breastfeeding practice in our sample (20, 21). These WHO-recommended approaches not only provide mothers with accessible channels for addressing breastfeeding difficulties but also support communities and healthcare systems in strengthening breastfeeding promotion and support.

While maternal PPD was not directly associated with EBF practice, our study identified a significant association between maternal PPD and negative intention to EBF. This finding is consistent with several previous studies (6, 7, 22, 23). Depressed mothers may experience sadness, hopelessness, and reduced confidence in infant care, which can lead to avoidance or reluctance to breastfeeding (11, 12, 24–26). In fact, a range of evidence-based interventions—such as home-visiting programs—has been shown to alleviate maternal emotional symptoms and improve the quality of feeding interaction, thereby promoting breastfeeding practice (21, 27). Moreover, recent studies have also indicated that breastfeeding may help alleviate postpartum depressive symptoms (28–30). Furthermore, the pathway analysis revealed a significant indirect effect, suggesting that maternal depression may influence breastfeeding behavior through its negative impact on mothers’ intention to EBF. According to the ABC behavioral theory, affective states (A) influence beliefs and cognitions (B), which in turn shape behavioral outcomes (C) (16). This framework has been widely applied to explain how emotional distress shapes health-related decision making and caregiving behaviors. In the context of breastfeeding, negative affective states such as sadness, hopelessness, and reduced self-efficacy may contribute to maladaptive cognitions about breastfeeding, thereby lowering motivation and consistency in maintaining EBF. Guided by this model, our findings suggest that maternal PPD (A) may undermine mothers’ intention to EBF (B), which may in turn affect their actual feeding practices (C). This finding emphasizing that improving mothers’ psychological well-being could help bridge the gap between breastfeeding intention and behavior.

Beyond maternal mental health, paternal psychological well-being also plays a crucial role in the family’s postpartum adjustment. New fathers may struggle to adapt to the transition to fatherhood and become vulnerable to depression due to factors such as negative life events, perceived stress, financial strain, and, notably, the presence of depressive symptoms in their partners (14, 31–34). Consistent with previous studies, our findings revealed a significant association between paternal and maternal PPD, underscoring the interdependence of parental mental health within families (32, 35–37). Building on this interdependence, paternal depression further indirectly affects maternal intention toward EBF by increasing maternal depressive symptoms. When fathers experience depression, their capacity to provide emotional support, childcare participation, and partner reassurance tends to decline. This reduction in paternal involvement may exacerbate maternal stress, decrease maternal confidence in breastfeeding, and, in severe cases, even impair lactation due to the mother’s heightened depressive symptoms (38–40). Moreover, fathers play a significant and independent role in shaping infants’ regulatory processes, including feeding (41). Evidence suggests that paternal depressive symptoms may interact with and exacerbate maternal emotional difficulties, resulting in greater mother–infant interaction conflict and heightened infant negative affectivity (42). These disruptions have been linked to an increased likelihood of non-breastfeeding feeding patterns, as emotionally strained triadic dynamics may reduce the feasibility of maintaining direct breastfeeding (43, 44). Such triadic dysregulation—spanning fathers, mothers, and infants—may ultimately create a less supportive emotional climate for breastfeeding, thereby undermining the establishment and maintenance of EBF. These findings highlight that PPD is not solely a maternal concern but a couple-level issue that can influence child-feeding outcomes. Routine PPD screening for both mothers and fathers should therefore be incorporated into standard postpartum follow-up and maternal–child health services. In addition, fathers may benefit from structured parenting support and psychological guidance to strengthen their ability to support breastfeeding. Actively involving fathers in postpartum mental health interventions and breastfeeding promotion programs may further contribute to successful exclusive breastfeeding.

A major strength of this study lies in identifying maternal intention as a key pathway linking parental depression to EBF practice. By emphasizing the pivotal role of intention in bridging intention and behavior, and by incorporating the mental health of both parents, this study offers a more comprehensive understanding of the psychological determinants of EBF. However, several limitations should be acknowledged. First, the cross-sectional design precludes causal inferences between PPD and EBF. Second, PPD was assessed using the EPDS rather than clinical diagnostic criteria, which may have led to an overestimation of its prevalence. Third, the analytic sample consisted of mothers with relatively higher education levels and better breastfeeding practices, which may introduce selection bias and limit the generalizability of our results; specifically, 56.8% of participants had attained a bachelor’s degree or higher, and 52.4% practiced exclusive breastfeeding. Future studies involving more diverse participants and improved data completeness are warranted. Finally, breastfeeding behavior is influenced by multiple unmeasured factors, such as parental knowledge of EBF, human resource and social welfare policies, and environmental or workplace support.

## Conclusion

This study revealed a substantial gap between mothers’ intention and actual practice of EBF. Maternal PPD was associated with intention to EBF but not with its practice, while paternal PPD showed an indirect association with EBF intention. These findings highlight the importance of assessing and addressing both maternal and paternal PPD to promote EBF. Future research and interventions should take a holistic approach, integrating individual, environmental, and policy factors to foster a breastfeeding-friendly society.

## Data Availability

The raw data supporting the conclusions of this article will be made available by the authors, without undue reservation.

## References

[1] Victora CG Bahl R Barros AJ Franca GV Horton S Krasevec J . Breastfeeding in the 21st century: epidemiology, mechanisms, and lifelong effect. Lancet. (2016) 387:475–90. doi: 10.1016/S0140-6736(15)01024-7, 26869575

[2] Lubis PN Saputra M Rabbani MW. A systematic review of the benefits of breastfeeding against postpartum depression in low-middle-income countries. J Ment Health. (2025) 34:305–17. doi: 10.1080/09638237.2024.2361232, 38869015

[3] Masi AC Stewart CJ. Role of breastfeeding in disease prevention. Microb Biotechnol. (2024) 17:e14520. doi: 10.1111/1751-7915.14520, 38946112 PMC11214977

[4] UNICEF. Breastfeeding: A mother’s gift, for every child. New York: (2018).

[5] UNICEF. Global breastfeeding scorecard, 2025. New York: (2025).

[6] Silva CS Lima MC Sequeira-de-Andrade LAS Oliveira JS Monteiro JS Lima NS . Association between postpartum depression and the practice of exclusive breastfeeding in the first three months of life. J Pediatr. (2017) 93:356–64. doi: 10.1016/j.jped.2016.08.005 28034730

[7] Woldeyohannes D Tekalegn Y Sahiledengle B Ermias D Ejajo T Mwanri L. Effect of postpartum depression on exclusive breast-feeding practices in sub-Saharan Africa countries: a systematic review and meta-analysis. BMC Pregnancy Childbirth. (2021) 21:113. doi: 10.1186/s12884-020-03535-1, 33557766 PMC7869485

[8] Henshaw EJ. Breastfeeding and postpartum depression: a review of relationships and potential mechanisms. Curr Psychiatry Rep. (2023) 25:803–8. doi: 10.1007/s11920-023-01471-3, 37906349

[9] Wang Z Liu J Shuai H Cai Z Fu X Liu Y . Mapping global prevalence of depression among postpartum women. Transl Psychiatry. (2021) 11:543. doi: 10.1038/s41398-021-01663-6 34671011 PMC8528847

[10] AI v d v d Z-B Boere-Boonekamp MM Groothuis-Oudshoorn CGM Reijneveld SA. Postpartum depression and anxiety: a community-based study on risk factors before, during and after pregnancy. J Affect Disord. (2021) 286:158–65. doi: 10.1016/j.jad.2021.02.062, 33725615

[11] Henshaw EJ Fried R Siskind E Newhouse L Cooper M. Breastfeeding self-efficacy, mood, and breastfeeding outcomes among Primiparous women. J Hum Lact. (2015) 31:511–8. doi: 10.1177/0890334415579654, 25829478

[12] Dubber S Reck C Muller M Gawlik S. Postpartum bonding: the role of perinatal depression, anxiety and maternal-fetal bonding during pregnancy. Arch Womens Ment Health. (2015) 18:187–95. doi: 10.1007/s00737-014-0445-4, 25088531

[13] Ahmadinezhad GS Karimi FZ Abdollahi M NaviPour E. Association between postpartum depression and breastfeeding self-efficacy in mothers: a systematic review and meta-analysis. BMC Pregnancy Childbirth. (2024) 24:273. doi: 10.1186/s12884-024-06465-4, 38609849 PMC11015580

[14] Rao WW Zhu XM Zong QQ Zhang Q Hall BJ Ungvari GS . Prevalence of prenatal and postpartum depression in fathers: a comprehensive meta-analysis of observational surveys. J Affect Disord. (2020) 263:491–9. doi: 10.1016/j.jad.2019.10.030, 31757623

[15] Le Bas G Aarsman SR Rogers A Macdonald JA Misuraca G Khor S . Paternal perinatal depression, anxiety, and stress and child development: a systematic review and Meta-analysis. JAMA Pediatr. (2025) 179:903–17. doi: 10.1001/jamapediatrics.2025.0880, 40522669 PMC12171964

[16] Dryden W Bond FW. Reason and emotion in psychotherapy: Albert Ellis. Br J Psychiatry. (1994) 165:131–5. doi: 10.1192/bjp.165.1.131, 7953024

[17] Lee DT Yip SK Chiu HF Leung TY Chan KP Chau IO . Detecting postnatal depression in Chinese women. Validation of the Chinese version of the Edinburgh postnatal depression scale. Br J Psychiatry. (1998) 172:433–7. 9747407 10.1192/bjp.172.5.433

[18] Guo XJ Wang YQ Liu Y Chen J Pu XF. Study on the optimal critical value of the Edinburgh postnatal depression scale in the screening of antenatal depression. Chin J Nurs. (2009) 44:808–10.

[19] Lai BPY Tang AKL Lee DTS Yip ASK Chung TKH. Detecting postnatal depression in Chinese men: a comparison of three instruments. Psychiatry Res. (2010) 180:80–5. doi: 10.1016/j.psychres.2009.07.015, 20493548

[20] WHO. Implementation guidance on counselling women to improve breastfeeding practices. Geneva: (2021).

[21] Vismara L Sechi C Lucarelli L. Reflective parenting home visiting program: a longitudinal study on the effects upon depression, anxiety and parenting stress in first-time mothers. Heliyon. (2020) 6:e04292. doi: 10.1016/j.heliyon.2020.e04292, 32671248 PMC7339064

[22] Annagur A Annagur BB Sahin A Ors R Kara F. Is maternal depressive symptomatology effective on success of exclusive breastfeeding during postpartum 6 weeks? Breastfeed Med. (2013) 8:53–7. doi: 10.1089/bfm.2012.0036, 23039400

[23] Fukui N Motegi T Watanabe Y Hashijiri K Tsuboya R Ogawa M . Exclusive breastfeeding is not associated with maternal-infant bonding in early postpartum, considering depression, anxiety, and parity. Nutrients. (2021) 13:1184. doi: 10.3390/nu13041184, 33918430 PMC8066877

[24] Vieira ES Caldeira NT Eugenio DS Lucca MMD Silva IA. Breastfeeding self-efficacy and postpartum depression: a cohort study. Rev Lat Am Enfermagem. (2018) 26:e3035. doi: 10.1590/1518-8345.2110.3035, 30208158 PMC6136553

[25] Stein A Pearson RM Goodman SH Rapa E Rahman A McCallum M . Effects of perinatal mental disorders on the fetus and child. Lancet. (2014) 384:1800–19. doi: 10.1016/s0140-6736(14)61277-0, 25455250

[26] Mercan Y Tari Selcuk K. Association between postpartum depression level, social support level and breastfeeding attitude and breastfeeding self-efficacy in early postpartum women. PLoS One. (2021) 16:e0249538. doi: 10.1371/journal.pone.0249538, 33798229 PMC8018654

[27] Ammaniti M Speranza AM Tambelli R Muscetta S Lucarelli L Vismara L . A prevention and promotion intervention program in the field of mother-infant relationship. Infant Ment Health J. (2006) 27:70–90. doi: 10.1002/imhj.20081, 28640423

[28] Lenells M Uphoff E Marshall D Wilson E Gustafsson A Wells MB . Breastfeeding interventions for preventing postpartum depression. Cochrane Database Syst Rev. (2025) 2:CD014833. doi: 10.1002/14651858.CD014833.pub2 39963955 PMC11834143

[29] Dessì A Pianese G Mureddu P Fanos V Bosco A. From breastfeeding to support in mothers' feeding choices: a key role in the prevention of postpartum depression? Nutrients. (2024) 16:2285. doi: 10.3390/nu16142285, 39064728 PMC11279849

[30] Rodríguez-Gallego I Vila-Candel R Corrales-Gutierrez I Gomez-Baya D Leon-Larios F. Evaluation of the impact of a midwife-led breastfeeding Group intervention on prevention of postpartum depression: a multicentre randomised clinical trial. Nutrients. (2024) 16:227. doi: 10.3390/nu16020227, 38257120 PMC10821517

[31] Duan Z Wang Y Jiang P Wilson A Guo Y Lv Y . Postpartum depression in mothers and fathers: a structural equation model. BMC Pregnancy Childbirth. (2020) 20:537. doi: 10.1186/s12884-020-03228-9, 32933502 PMC7493423

[32] Glasser S Lerner-Geva L. Focus on fathers: paternal depression in the perinatal period. Perspect Public Health. (2019) 139:195–8. doi: 10.1177/1757913918790597, 30044191

[33] Wang D Li YL Qiu D Xiao SY. Factors influencing paternal postpartum depression: a systematic review and Meta-analysis. J Affect Disord. (2021) 293:51–63. doi: 10.1016/j.jad.2021.05.088, 34171611

[34] Kitil GW Hussen MA Chibsa SE Chereka AA. Exploring paternal postpartum depression and contributing factors in Ethiopia: a systematic review and meta-analysis. BMC Psychiatry. (2024) 24:754. doi: 10.1186/s12888-024-06206-z, 39478469 PMC11526678

[35] Zhao XH Zhang ZH. Risk factors for postpartum depression: an evidence-based systematic review of systematic reviews and meta-analyses. Asian J Psychiatr. (2020) 53:102353. doi: 10.1016/j.ajp.2020.102353, 32927309

[36] Paulson JF Bazemore SD. Prenatal and postpartum depression in fathers and its association with maternal depression: a meta-analysis. JAMA. (2010) 303:1961–9. doi: 10.1001/jama.2010.605, 20483973

[37] Smythe KL Petersen I Schartau P. Prevalence of perinatal depression and anxiety in both parents: a systematic review and Meta-analysis. JAMA Netw Open. (2022) 5:e2218969. doi: 10.1001/jamanetworkopen.2022.18969, 35749112 PMC9233234

[38] Paulson JF Dauber S Leiferman JA. Individual and combined effects of postpartum depression in mothers and fathers on parenting behavior. Pediatrics. (2006) 118:659–68. doi: 10.1542/peds.2005-2948, 16882821

[39] Goodman JH. Paternal postpartum depression, its relationship to maternal postpartum depression, and implications for family health. J Adv Nurs. (2004) 45:26–35. doi: 10.1046/j.1365-2648.2003.02857.x, 14675298

[40] Shimao M Matsumura K Tsuchida A Kasamatsu H Hamazaki K Inadera H . Influence of infants' feeding patterns and duration on mothers' postpartum depression: a nationwide birth cohort -The Japan Environment and children's Study (JECS). J Affect Disord. (2021) 285:152–9. doi: 10.1016/j.jad.2021.02.011, 33667755

[41] Lamb ME Lewis C. Father-child relationships. In: Cabrera NJ Tamis-LeMonda CS editors. Handbook of father involvement. New York: Routledge (2013) 119–34.

[42] Lucarelli L Vismara L Chatoor I Sechi C. Parental pre and postnatal depression: The longitudinal associations with child negative affectivity and dysfunctional mother-child feeding interactions. Children (Basel). (2023) 10:565. doi: 10.3390/children10030565, 36980124 PMC10047090

[43] Ventura A Hupp M Lavond J. Mother-infant interactions and infant intake during breastfeeding versus bottle-feeding expressed breast milk. Matern Child Nutr. (2021) 17:e13185. doi: 10.1111/mcn.13185, 33939269 PMC8476436

[44] Lavelli M Poli M. Early mother-infant interaction during breast-and bottle-feeding. Infant Behav Dev. (1998) 21:667–83. doi: 10.1016/s0163-6383(98)90037-6

